# Dihydromyricetin Inhibits M1 Macrophage Polarization in Atherosclerosis by Modulating miR-9-Mediated SIRT1/NF-*κ*B Signaling Pathway

**DOI:** 10.1155/2023/2547588

**Published:** 2023-05-17

**Authors:** Zhousheng Yang, Tianyu Li, Chunyan Wang, Mingyu Meng, Shenglan Tan, Lei Chen

**Affiliations:** ^1^Department of Pharmacy, The People's Hospital of Guangxi Zhuang Autonomous Region, Guangxi Academy of Medical Sciences, Nanning 530021, China; ^2^Department of Pharmacy, The Second Xiangya Hospital, Central South University, Changsha, China 410011; ^3^Institute of Clinical Pharmacy, Central South University, Changsha, China 410011

## Abstract

Dihydromyricetin (DMY), a natural flavonoid compound extracted from the stems and leaves of *Ampelopsis grossedentata*, has been found as a potential therapeutic chemical for treating atherosclerosis. This study explores the underlying mechanism of DMY repressing M1 macrophage polarization in atherosclerosis. We showed that DMY treatment markedly decreased M1 macrophage markers (e.g., Tnf-*α* and IL-1*β*) and p65-positive macrophage numbers in the vessel wall of Apoe-deficient (*Apoe^–/–^*) mice. Overexpression of miR-9 or knockdown of SIRT1 in macrophages reversed the effect of DMY on M1 macrophage polarization. The data we presented in the study indicate that the miR-9-mediated SIRT1/NF-*κ*B pathway plays a pivotal role in M1 macrophage polarization and is one of the molecular mechanisms underlying the anti-atherosclerosis effects of DMY. We provide new solid evidence that DMY may be explored as a potential therapeutic adjuvant for treating atherosclerosis.

## 1. Introduction

Cardiovascular diseases (CVDs) are the leading cause of mortality worldwide, responsible for one-third of all-cause deaths [1, 2]. The accumulative formation of unstable atherosclerosis plaque in the vessel wall has been recognized as a hallmark in the progress of cardiovascular diseases [3, 4]. Enormous evidence from clinical trials and fundamental researches has demonstrated that atherosclerosis is a lipid-driven chronic inflammatory disease [5–7]. Macrophages, one of the most important immune cells in inflammation, are divided into two subtypes based on their biological functions [8]. One phenotype is M1-like macrophages (classically activated), which secrete inflammatory cytokines (e.g., TNF-*α* and IL-1*β*) and promote inflammation. The other one is M2-like macrophages (alternatively activated), which produce anti-inflammatory cytokines (e.g., IL-10 and Arg1) and repair injured tissues. Abnormal activation and infiltration of macrophages in the vessel wall have been identified as a crucial character of unstable plaque. More specifically, increased M1 macrophages with decreased M2 macrophages in the lesion aggravate the vascular inflammation and eventually accelerate atherosclerosis [9, 10].

Dihydromyricetin (DMY), a natural flavonoid compound extracted from the stems and leaves of *Ampelopsis grossedentata*, has been reported to exert anti-inflammatory activities in various diseases [11, 12]. For instance, DMY declines IL-1*β* and TNF-*α* levels in the LPS-treated rats' plasma via inhibiting oxidative stress [13]. DMY was also found to inhibit systemic inflammation and prevent infiltration of inflammatory cells in the joints, resulting in relief of synovitis in rheumatoid arthritis [14, 15]. Most recently, Zhou et al. [16] reported that in an exhaustive exercise- (EE-) induced hepatic inflammatory injury animal model, DMY suppressed hepatic inflammation by orchestrating macrophage polarization. In agreement with these findings, we also observed that DMY decreased the expression of M1 macrophage markers (e.g., Tnf-*α* and IL-1*β*) and macrophage accumulation in the vessel wall of *Apoe^–/–^* mice [17]. However, the underlying mechanisms about how DMY regulates macrophage polarization and alleviates vascular inflammation are largely unknown.

MicroRNAs (miRNAs), a class of small noncoding RNA, have been recognized as potent regulators of various pathological and physiological processes by binding to the 3′UTR of target mRNA [18, 19]. Recently, multiple studies have indicated that miR-9, a highly expressed miRNA in M1 macrophage, plays an essential role in promoting M1 polarization by targeting SIRT1/NF-*κ*B signal pathway activation [20, 21]. In this study, we found that DMY downregulated miR-9 and upregulated its target gene SIRT1 expression both in vivo and in vitro. We identified the role of the miR-9/SIRT1/NF-*κ*B signaling pathway on the antiatherosclerosis effect of DMY, which may, through regulating macrophage polarization and subsequently alleviating vascular inflammation. This study provides new evidence that DMY may emerge as a potential therapeutic adjuvant in treating atherosclerosis.

## 2. Materials and Methods

### 2.1. Animal Protocol

All animal procedures were approved by the Institutional Animal Care and Use Committee of Second Xiangya Hospital, Central South University. *Apoe^–/–^* mice and C57BL/6J mice of 8~12 weeks old were purchased from the Beijing Vital River Laboratory Animal Technology Co. and were fed on a chow diet or a high cholesteric diet (HCD) (D12108C, Research Diets, USA) for 12 weeks. *Apoe^–/–^* mice were administered daily with DMY (500 mg/kg; D101549, Aladdin; *n* = 8) or the solvent of 0.05% CMC-Na (*n* = 10) via intragastric gavage. The liver, aorta, and peripheral blood mononuclear cells (PBMC) were harvested after 12 weeks. Aortic roots were embedded in an optimal cutting temperature (OCT) compound and stored at -80°C.

### 2.2. Immunofluorescent Staining

The detail of immunofluorescent staining was described in our previous study [17]. Briefly, the OCT-embedded aorta root was cut into sections at 6 *μ*m using a Lab-Tek tissue processor Leica CM1950. Sections were incubated with anti-Mac-2 (1 : 100, #CL8942AP, Cedarlane) and anti-p65 (1 : 200 dilution, Thermo Fisher Scientific) at 4°C overnight, followed by being stained with Alexa Flour 555 (1 : 300, #A21434, Invitrogen) or 488 (1 : 300, #A11034, Invitrogen) for 1 hour at room temperature. DAPI (#P36935, Invitrogen) was used to label the nuclei. All images were collected by confocal laser scanning microscopy (Nikon, Japan).

### 2.3. Primary Macrophage Culture, Stimulation, and Transfection

Bone marrow-derived macrophages (BMDMs) were extracted from 12-week-old C57 BL/6J mice. BMDMs were cultured in DMEM medium containing 10% FBS and 10 ng/ml Recombinant Mouse M-CSF Protein (416-ML, R&D), followed by treatment with DMY (50, 100 *μ*mol/L) or control vehicle for one hour, then stimulated with 100 ng/ml LPS (Cat# L2880, Sigma-Aldrich) and 20 ng/ml INF-*γ* (IF005, Sigma-Aldrich) for 24 hours. Lipofectamine 3000 transfection reagent (L3000008, Invitrogen) was used for miRNA (miR10000142-1-5, miR20000142-1-5, RiboBio) and siRNA (AM16708, Thermo Fisher Scientific) transfection according to the manufacturers' instructions.

### 2.4. Real-Time qPCR

Total RNA was isolated by TRIzol reagent (15596018, Invitrogen) from the homogenized liver, aorta, and BMDMs. The PrimeScript RT reagent kit (RR047A, Takara) was used to generate cDNA, and the TB Green Premix EX Tag kit (RR820A, Takara) was used for real-time qPCR with the Real-time PCR system (Roche) following the manufacturer's instructions. Specific primers, including miR-9 and U6 (MQPS0000002-1-100), were purchased from RiboBio (Guangzhou, China). Primers' information is listed as follows: *β*-actin (forward: 5′-GAAATCGTGCGTGACATCAAAG-3′, reverse: 5′-TGTAGTTTCATGGATGCCACAG-3′); IL-1*β* (forward: 5′-GCAACTGTTCCTGAACTCAACT-3′, reverse: 5′-ATCTTTTGGGGTCCGTCAACT-3′); IL-6 (forward: 5′-TAGTCCTTCCTACCCCAATTTCC-3′, reverse: 5′-TTGGTCCTTAGCCACTCCTTC-3′); Tnf-*α* (forward: 5′-CCCTCACACTCAGATCATCTTCT-3′, reverse: 5′-GCTACGACGTGGGCTACAG-3′); Nos2 (forward: 5′-GTTCTCAGCCCAACAATACAAGA-3′, reverse: 5′-GTGGACGGGTCGATGTCAC-3′); IL-10 (forward: 5′-GCTCTTACTGACTGGCATGAG-3′, reverse: 5′-CGCAGCTCTAGGAGCATGTG-3′); Arg1 (forward: 5′-GTGGAAACTTGCATGGACAAC-3′, reverse: 5′-AATCCTGGCACATCGGGAATC-3′); and SIRT1 (forward: 5′-GACGCTGTGGCAGATTGTTA-3′, reverse: 5′-AAACATGGCTTGAGGGTCTG-3′).

### 2.5. Western Blot

Protein samples were extracted and measured using a BCA kit (Beyontime, Jiangsu, China). Target protein (20 *μ*g of each sample) was separated by 10% SDS-PAGE gels and then transferred onto 0.45 *μ*m polyvinylidene fluoride (PVDF) membranes (Millipore, Billerica, MA, USA) and followed by incubation with 5% fat-free milk in TBST for 90 min at RT and subsequently interacted with corresponding antibodies as follows: p-p65 and SIRT1 (1 : 1000, Abcam, USA), IkB*α* (1 : 1000, Cell Signaling Technology, USA), and *β*-actin (1 : 5000, Sigma, USA) at 4°C overnight. The images were captured using the Bio-Rad Chemi Doc XRS+ Imaging System (Bio-Rad Biosciences, USA).

### 2.6. Statistical Analysis

All data were analyzed using SPSS 26.0 statistical software (IBM Corp., NY, USA), and GraphPad Prism 8.0 (GraphPad Software, CA, USA) was used for data present in the study. Unpaired two-tailed Student's *t*-test was used to calculate statistical significance between two groups for normally distributed continuous variables. One-way analysis of variance (ANOVA) was utilized for multiple group comparison. Nonparametric Mann–Whitney *U* test or Kruskal-Wallis test was used for data without normal distribution. All data were expressed as mean ± SEM, and ^∗^*P* < 0.05, ^∗∗^*P* < 0.01, and ^∗∗∗^*P* < 0.001 were considered significant for all tests.

## 3. Results

### 3.1. DMY Decreases Expression of M1 Markers and Increases Expression of M2 Markers in Apoe^−/−^ Mice and LPS-Stimulated BMDMs

**Figure 1:**
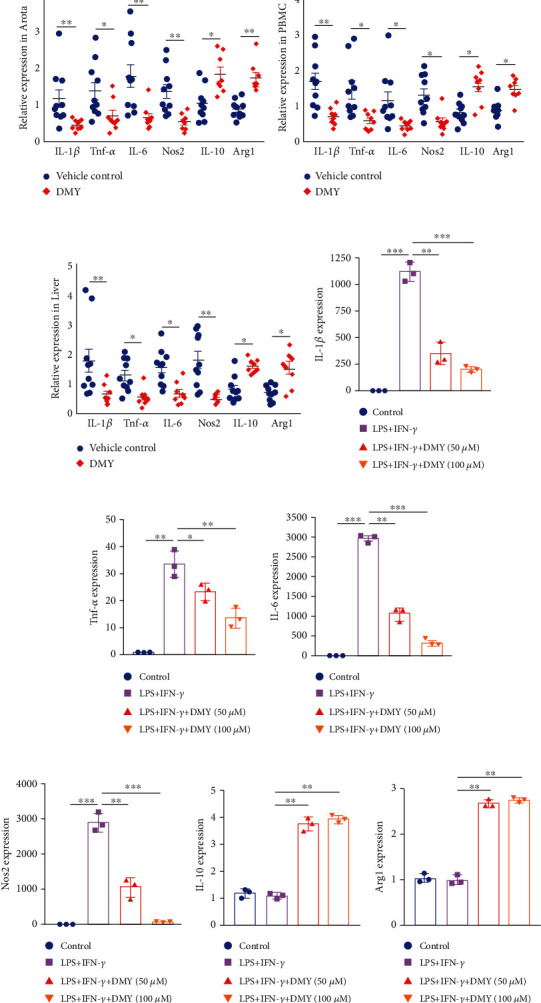
(a–c) qPCR analysis of the expression of M1/M2 markers in the tissues between the vehicle control (*n* = 10) and DMY (*n* = 8) groups. (d–i) qPCR verification M1/M2 marker expression in LPS-induced BMDMs with/without DMY treatment (*n* = 3). ^∗^*P* < 0.05, ^∗∗^*P* < 0.01, and ^∗∗∗^*P* < 0.001.

To investigate whether macrophage polarization is a potential antiatherosclerosis target of DMY, we performed several experiments with the tissues of the aorta, PBMC, and liver from the *Apoe^–/–^* mice harvested in our previous study [17]. As shown in Figures 1(a)–1(c), compared with vehicle-treated *Apoe^–/–^* mice, M1 markers' expressions (IL-1*β*, Tnf-*α*, IL-6, and Nos2) in the tissues were downregulated and expressions of M2 markers (IL-10 and Arg1) were upregulated in the DMY-treated *Apoe^–/–^* mice. In line with the in vivo data, qPCR showed that expressions of M1 markers dramatically declined in LPS-stimulated BMDMs with the DMY treatment in a dose-dependent manner (Figures 1(d)–1(g)). Interestingly, elevated M2 marker expressions in the BMDMs with DMY treatment were not significantly different between the high concentration and low concentration of the DMY group (Figures 1(h) and 1(i)). Taken together, our in vivo and in vitro data demonstrate that DMY inhibits M1 macrophage polarization and promotes M2 macrophage polarization in atherosclerosis.

### 3.2. DMY Suppresses M1 Macrophage Polarization via miR-9 in BMDMs

**Figure 2:**
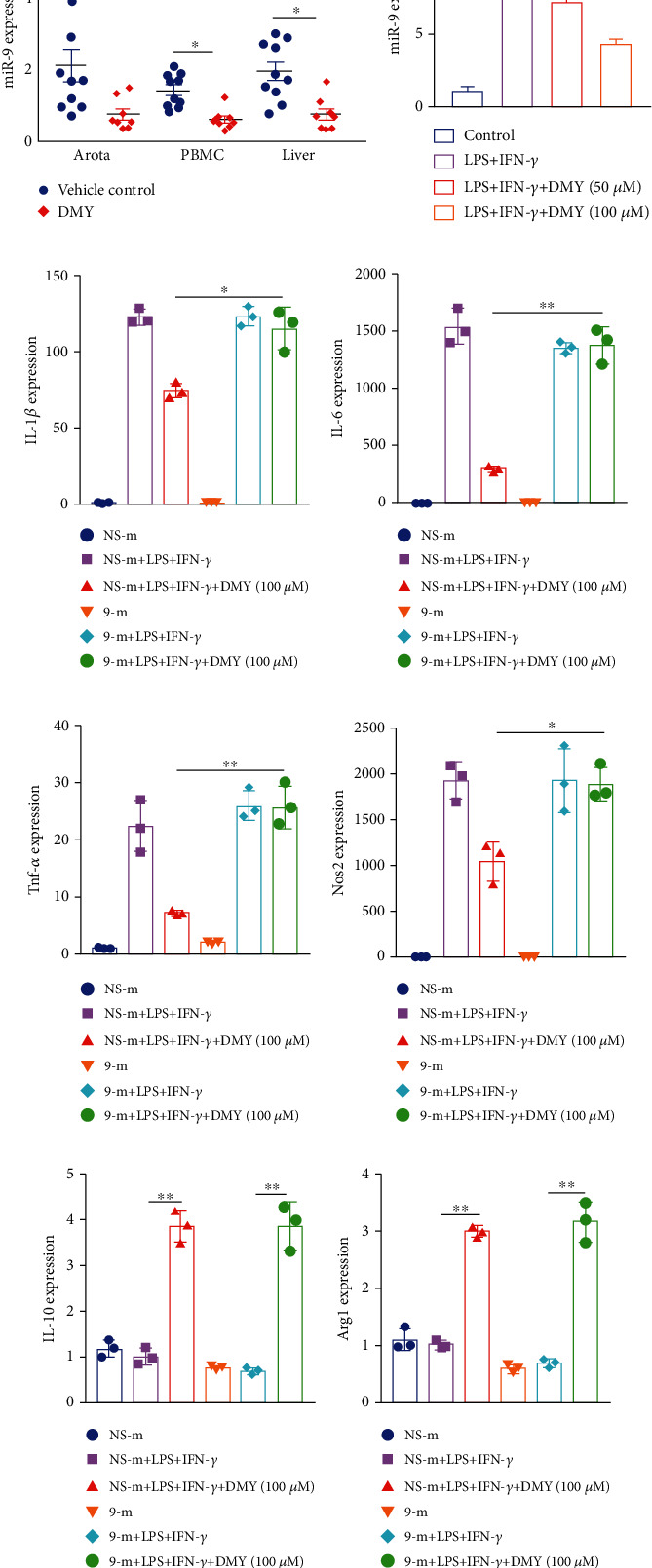
(a) The expression of miR-9 in the vehicle control (*n* = 10) and DMY (*n* = 8) groups. (b) qPCR detection of miR-9 expression in BMDMs in each group (*n* = 3). (c–h) Analysis of M1/M2 markers after mimic Nc and miR-9 transfection in treated BMDMs in each group (*n* = 3). ^∗^*P* < 0.05 and ^∗∗^*P* < 0.01.

Numerous studies have indicated that miR-9 plays a crucial role in macrophage polarization in inflammatory diseases [22, 23]. Therefore, we next explored whether miR-9 was associated with DMY's antiatherosclerosis effect. We investigated the expression of miR-9 and found that miR-9 was decreased in the aorta, PBMC, and liver of *Apoe^–/–^* mice after intragastric gavage with DMY (Figure 2(a)). Consistent with the in vivo data, miR-9 expression was significantly repressed by DMY treatment in the LPS-stimulated BMDMs (Figure 2(b)). Transfection of miR-9 mimic was used to validate this hypothesis. As we expected, the downregulation of IL-1*β*, Tnf-*α*, IL-6, and Nos2 expressions was reversed by miR-9 transfection in the DMY group (Figures 2(c)–2(f)). To our surprise, increased expression of IL-10 and Arg1 in BMDMs with DMY treatment could not be reversed by miR-9 transfection (Figures 2(g) and 2(h)). In summary, these results indicate that overexpression of miR-9 could abrogate the effect of DMY on M1 macrophage polarization but not M2 macrophages.

### 3.3. miR-9 Mediates the Effect of DMY on M1 Macrophage Polarization by Targeting SIRT1/NF-*κ*B Signaling Pathway in BMDMs

**Figure 3:**
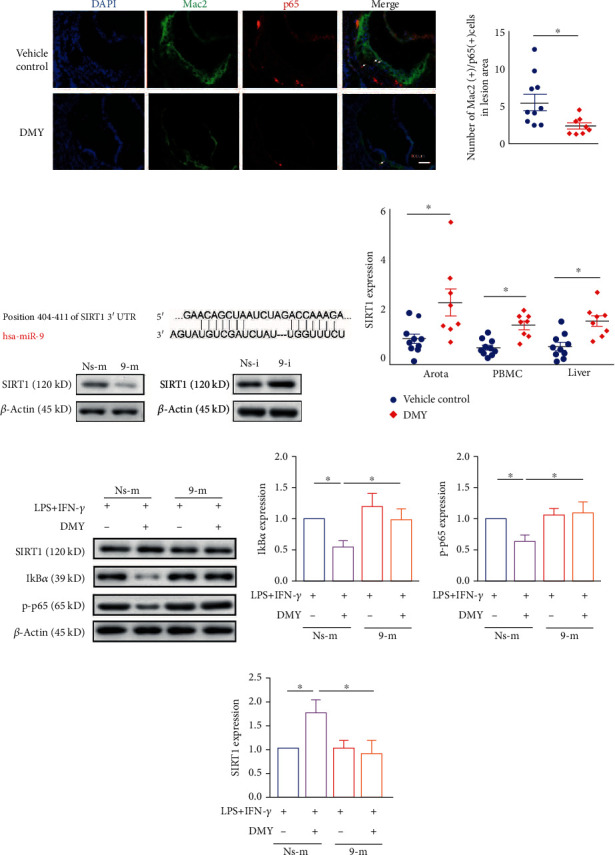
(a) Representative images are shown p65 colocalized with Mac2 in the aortic sinus lesions of the vehicle control and DMY groups. Scale bar, 100 *μ*m. (b) The binding site between miR-9 and SIRT1 was predicted by the TargetScan database. (c) qPCR examines the expression of SIRT1 in the tissues. (d–g) Western blot analysis of NF-*κ*B signaling pathway in BMDMs in each group (*n* = 3). ^∗^*P* < 0.05.

The abnormal activation of the NF-*κ*B signaling pathway is a core factor in M1 macrophage polarization and the pathogenesis of atherosclerosis. The NF-*κ*B signaling pathway has been reported as a potential pharmacological target for DMY [24–26]. As shown in Figure 3(a), we found that DMY diminished Mac2 positive/p-65 positive cell numbers in the aortic sinus lesions of *Apoe^–/–^* mice. How does DMY affect macrophage polarization and eventually ameliorate atherosclerosis? Recently, Wang et al. [21] revealed that miR-9 promoted M1 macrophage polarization in osteoarthritis progression by targeting SIRT1 and subsequently activating the NF-*κ*B signal pathway. In agreement with Wang et al.'s study, we found that SIRT1 was a direct target of miR-9 in macrophages (Figure 3(b)). Moreover, elevated SIRT1 expression was observed in *Apoe^–/–^* mice with DMY treatment, which indicated that the miR-9/SIRT1 pathway may contribute to the regulation of DMY on M1 macrophage polarization (Figure 3(c)). Thus, we transfected miR-9 mimic in BMDMs and found that miR-9 transfection abolished the increased expression of SIRT1 and inhibited the NF-*κ*B signaling pathway under the treatment of DMY (Figures 3(d)–3(g)). The data mentioned above indicate that DMY suppresses M1 macrophage polarization, probably by targeting the miR-9/SIRT1/NF-*κ*B signaling pathway.

### 3.4. The Effect of DMY on M1 Macrophage Polarization Depends on the SIRT1/NF-*κ*B Signaling Pathway in BMDMs

**Figure 4:**
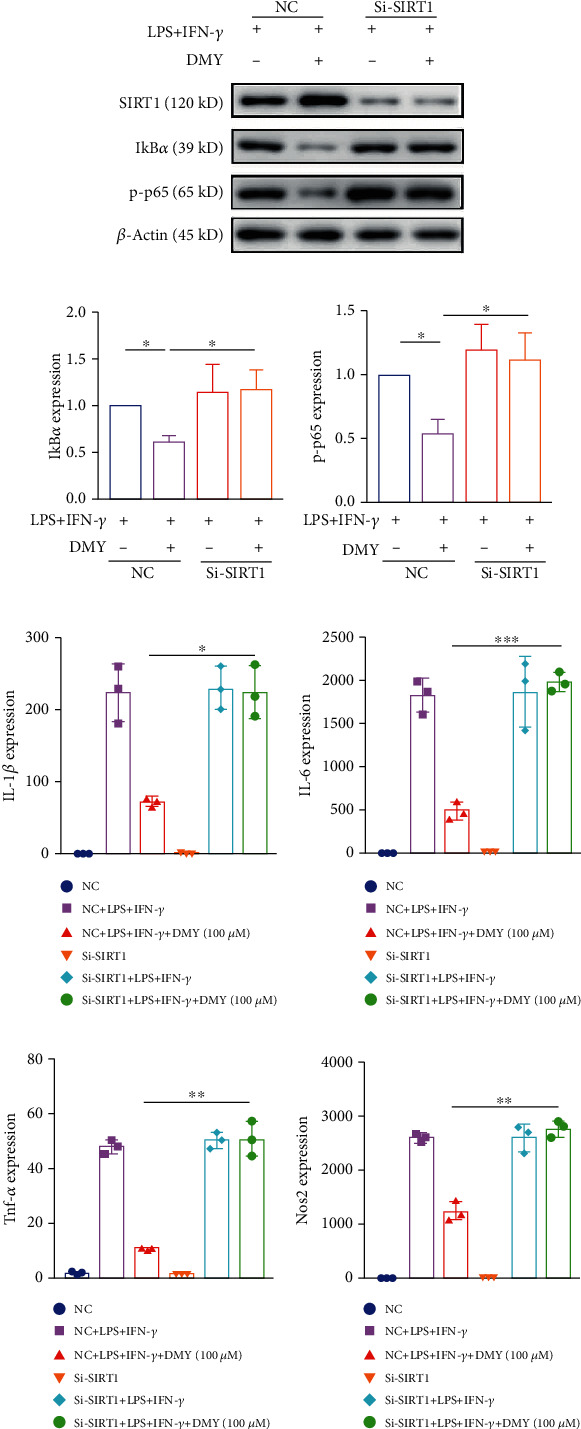
(a–c) Western blot detects SIRT1 and NF-*κ*B signaling pathway in each group (*n* = 3). (d–g) qPCR examines the expression of M1 markers in BMDMs in each group (*n* = 3). ^∗^*P* < 0.05, ^∗∗^*P* < 0.01, and ^∗∗∗^*P* < 0.001.

To confirm whether SIRT1 was involved in the effect of DMY on M1 macrophage polarization, siRNA was used to knock down the expression of SIRT1 in BMDMs. We found that silence of SIRT1 rescued the decrease of IkB*α* and p-p65 in BMDMs treated with DMY (Figures 4(a)–4(c)); meanwhile, the declined expressions of M1 markers (IL-1*β*, Tnf-*α*, IL-6, and Nos2) in the DMY group were also reversed (Figures 4(d)–4(g)). These results reveal that SIRT1 is the crucial factor contributing to the effect of DMY on M1 macrophage polarization by modulating the NF-*κ*B signaling pathway.

## 4. Discussion

Accumulating clinical and experimental studies have demonstrated that traditional Chinese herbal medicine is a promising therapy for cardiovascular diseases [27, 28]. Here, we provided evidence that DMY relieved vascular inflammation and repressed M1 macrophage polarization in atherosclerosis through modulating the miR-9/SIRT1/NF-*κ*B signal pathway. Overexpression of miR-9 or knockdown of SIRT1 expression in macrophage could both reverse the effect of DMY on M1 macrophage polarization. The data we presented above indicate that the miR-9-mediated SIRT1/NF-*κ*B pathway plays a pivotal role in M1 macrophage polarization and is one of the molecular mechanisms underlying the antiatherosclerosis effects of DMY.

Macrophages are involved in all stages of atherosclerosis, from lesion initiation to rupture of advanced lesions. More specifically, M1 and M2 macrophages are recruited to the intima at an early stage to eliminate accumulated lipids and repair injured tissue. In the advanced stage, cumulative M1 macrophages and decreased M2 macrophages appear in the lesion, leading to the formation of a necrotic core which may result in a cardiovascular event [29, 30]. Targeting macrophage polarization is considered one of the most promising therapeutic strategies for atherosclerosis [31]. Recently, Zhou et al. [16] found that DMY-encapsulated liposomes efficiently inhibited exercise-induced liver inflammation by targeting hepatic macrophages, repressing M1 macrophage polarization, and promoting M2 macrophage polarization. Our previous study also reported that DMY inhibited macrophage accumulation in the aortic sinus lesions and liver of *Apoe^–/–^* mice [17]. In the present study, decreased expressions of M1 markers and increased expressions of M2 markers in the circulating monocytes, livers, and aorta were observed in DMY-treated *Apoe^–/–^* mice (Figures 1 and 3(a)). Our data suggested that M1/M2 polarization may be an essential target for DMY to alleviate vascular inflammation and eventually ameliorate atherosclerosis.

How does DMY regulate M1/M2 polarization? Numerous studies have demonstrated that highly expressed miR-9 in macrophage induced by pathological stimulus (e.g., LPS) during the inflammatory response is associated with M1 polarization [32, 33]. Tong et al. [20] showed that head and neck squamous cell carcinoma- (HNSCC-) derived exosomal miR-9 could induce M1 macrophage polarization. In support, another study [34] found that lipotoxic hepatocyte-derived extracellular vesicle- (EV-) encapsulated miR-9-5p markedly activated hepatic macrophage M1 polarization both in vivo and in vitro. These researches indicate that miR-9 secreted and delivered from macrophages or other cell types can induce M1 macrophage polarization. Thus, we hypothesized that miR-9 was a potential target of DMY in macrophages, which mediated the effect of DMY on macrophage polarization. As we expected, downregulation of miR-9 expression was observed in DMY-treated *Apoe^–/–^* mice and BMDMs (Figures 2(a) and 2(b)). More critically, overexpression of miR-9 by miR-9 mimic in BMDMs entirely blocked the inhibition effect of DMY on M1 macrophage polarization (Figures 2(c)–2(f)). Surprisingly, DMY-induced increased expressions of M2 markers in macrophage cannot be abrogated by miR-9 overexpression (Figures 2(g) and 2(h)). Our data indicated that miR-9 mediated the regulation of DMY on M1 macrophage polarization but not M2 macrophage polarization. The underlying molecular mechanisms of DMY on M2 macrophage polarization require further investigation.

Impaired expression of SIRT1 promotes p65 nucleus translocation in macrophages, resulting in the phenotype switching to M1 [35]. An increasing number of data showed that traditional Chinese herbs, including DMY, alleviated inflammation by modulating M1/M2 polarization in a SIRT1/NF-*κ*B pathway-dependent manner. Zeng et al. [36] recently reported that DMY treatment increased hepatic SIRT1 expression and subsequently repressed the NF-*κ*B signal pathway in nonalcoholic steatohepatitis (NASH) mice. Consistent with Zeng et al.'s finding, we found that DMY elevated SIRT1 expression in *Apoe^−/−^* mice and BMDMs (Figures 3(c) and 3(d)). More importantly, overexpression of miR-9 repressed the increased expression of SIRT1 in DMY-treated BMDMs (Figure 3(d)). Knockdown of SIRT1 in macrophages promoted NF-*κ*B pathway activation and abrogated the suppression of DMY on M1 macrophage polarization (Figure 4). Taken together, we hypothesized that DMY modulated SIRT1 expression by decreasing the expression of miR-9, which can inhibit NF-*κ*B pathway activation and M1 macrophage polarization. Of note, Zhou et al.'s [16] finding revealed that SIRT3 was the critical target for DMY-encapsulated liposomes to orchestrate M1/M2 macrophage polarization and improve exhaustive exercise-induced hepatic inflammation. However, our study did not investigate if SIRT3 played a role in the antiatherosclerosis effect of DMY, which will be examined in future studies.

In this study, DMY alleviated vascular inflammation by orchestrating M1/M2 macrophage polarization. Zeng et al. [37] also revealed that DMY facilitates macrophage cholesterol efflux and prevents foam cell formation in an LXR*α*-ABCA1/ABCG1-dependent manner. Moreover, we [17] and Liu et al. [38] confirmed that DMY increased endothelial nitric oxide production, improved lipid profiles, downregulated hepatic inflammation, and inhibited atherosclerosis. In addition, DMY could modulate gut microbiota to either improve DSS-induced colitis or exert an antiobesity effect [39, 40]. These previous findings indicate that gut microbiota is a potential target for DMY in treating atherosclerosis. Collectively, DMY exerts antiatherosclerosis activities by targeting multiple cell types, tissues, and gut microbiota involved in the pathological process of atherogenesis. Herein, in the present data, we highlight that orchestrating M1/M2 polarization may be an essential target for DMY to alleviate vascular inflammation and ameliorate atherosclerosis. Furthermore, the miR-9-mediated SIRT1/NF-*κ*B pathway is a crucial target for DMY to inhibit M1 macrophage polarization.

There are several limitations in our research. Firstly, our data from the in vivo samples (aorta, PBMC, and liver) was based on mixed cell populations, and macrophage-specific miR-9 knockout/in mice are needed to validate our hypothesis further. Secondly, miR-9 mediated the effect of DMY on M1 macrophage polarization but not M2 macrophage polarization, which indicates that other regulation mechanisms may contribute to the impact of DMY on orchestrating macrophage polarization.

## 5. Conclusion

**Figure 5:**
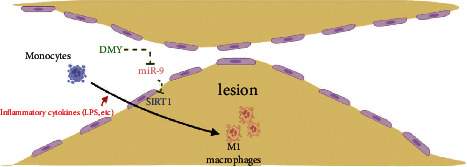
Scheme of miR-9 mediates the inhibition of DMY on M1 macrophage polarization in atherosclerosis.

In summary, our data demonstrate that miR-9 contributes to the inhibition effect of DMY on M1 macrophage polarization, at least in part, by targeting SIRT1 and activating the NF-*κ*B pathway in atherosclerosis (Figure 5). Our findings shed new insights on how DMY regulates macrophage activation and provides solid evidence that DMY may be explored as a potential therapeutic adjuvant in treating atherosclerosis.

## Data Availability

The datasets used and/or analyzed during the current study are available from the corresponding authors upon reasonable request.
